# Copper-coated carbon-infiltrated carbon nanotube surfaces effectively inhibit *Staphylococcus aureus* and *Pseudomonas aeruginosa* biofilm formation

**DOI:** 10.1128/aem.01053-25

**Published:** 2025-07-08

**Authors:** Lucy C. Bowden, Sidney T. Sithole, Emilia C. Walton, Jun Han Chen, Jason J. Sorensen, Anton E. Bowden, Brian D. Jensen, Bradford K. Berges

**Affiliations:** 1 Department of Microbiology and Molecular Biology, Brigham Young University 6756, Provo, Utah, USA; 2 Department of Chemistry and Biochemistry, Brigham Young University 6756, Provo, Utah, USA; 3 Department of Mechanical Engineering, Brigham Young University 6756, Provo, Utah, USA; INRS Armand-Frappier Sante Biotechnologie Research Centre, Laval, Quebec, Canada

**Keywords:** biofilm, *Staphylococcus aureus*, MRSA, copper, carbon nanotubes, nanostructured surface

## Abstract

Implant-associated infections caused by *Staphylococcus aureus* are a growing problem for healthcare systems. Implant materials that resist bacterial colonization may help reduce infection rates and severity. This research examined the effect of a copper-coated carbon-infiltrated carbon nanotube surface (Cu-CICNT). We have previously shown that CICNT without copper has an anti-biofilm effect, and copper has long been known to have anti-bacterial properties. Bacterial biofilms were grown in a droplet on the Cu-CICNT surface, and a control consisting of copper deposited on a relatively flat, non-nanotube-structured surface. The Cu-CICNT surface was highly effective at reducing biofilm formation, reducing recoverable *S. aureus* bacteria by 99.9999% in 12 hours (a 6.3-log reduction). This effect was confirmed in both a methicillin-resistant and a methicillin-sensitive isolate of *S. aureus*. The Cu-CICNT surface was also highly effective against *Pseudomonas aeruginosa*, resulting in a 6.9-log reduction in adherent bacteria. The Cu-CICNT surface was more effective at inhibiting biofilm formation than the flat copper-coated titanium, indicating a synergistic effect between the CICNT topography and copper. The concentration of copper ions in growth media was low after exposure to Cu-CICNT (6.2 ppm), and media with this amount of supplemented copper had only a small effect on biofilm reduction, as did conditioned media previously exposed to Cu-CICNT. Our findings suggest that the antibacterial effect is likely due to contact killing of bacteria on the textured copper surface.

**IMPORTANCE:**

Orthopedic implants and devices are becoming increasingly common. Unfortunately, as their use increases, so does the prevalence of implant-associated infections. These infections are most commonly caused by the bacterium *Staphylococcus aureus. S. aureus* infections are particularly difficult to treat because they form biofilms resistant to antibiotics and the host immune system. In this research, we used a carbon nanotube-based surface combined with a thin film of copper to produce a surface coating that could be used on implants to prevent bacterial infection. The combination of the surface topography with the copper coating resulted in over a 6-log reduction in the number of adherent bacteria, preventing the formation of a bacterial biofilm. This reduction in adherent bacteria is likely due to the surface killing effects of the bacteria on contact. The potential applications of such a surface could help reduce infection burden, improve patient quality of life, and reduce stress on healthcare systems.

## INTRODUCTION

Orthopedic implants and prosthetic devices greatly improve patient quality of life and movement capacity. Their use is increasing, with over one million hip and knee replacements occurring each year in the United States alone (1). Infections following such surgeries affect 0.5%–2% of all joint arthroplasty (2, 3) and are a challenging problem. Recalcitrant implant-associated infections are often treated with a two-step revision surgery. Infections account for about 23% of all-cause revision surgeries (4). Although necessary and considered the standard of care, these two-step revision surgeries are associated with a 5-year mortality rate of over 20% (5, 6).

Implant-associated infections are most frequently caused by staphylococci, particularly *Staphylococcus aureus* (7). *S. aureus* is a gram-positive bacterium commonly found in healthcare settings that can cause osteomyelitis, bacteremia, and other infections (8). Other bacterial species that can cause implant-associated infections include *Streptococcus*, *Enterobacteriaceae*, and *Pseudomonas* species (7, 9). While the majority of implant-associated infections are caused by *S. aureus* (7)*,* infections caused by *Pseudomonas aeruginosa* are considered particularly difficult to treat due to the high frequency of antibiotic-resistant bacteria (9). Both *S. aureus* and *P. aeruginosa* form biofilms, surface-associated assemblages of bacteria that are extremely difficult to remove or treat via the host immune response or antibiotic treatment. In addition, many isolates of *S. aureus* and *P. aeruginosa* are resistant to antibiotics, and the biofilm environment increases bacterial antibiotic resistance (10). Alternatives to antibiotic treatment are badly needed. One alternative method to fight implant-associated infections could be implant surfaces designed with inherent antibacterial and anti-biofilm properties. This could be achieved through the use of nanotextured surfaces that prevent biofilm growth or through chemical means using antimicrobial compounds on the implant surface.

The topography of nanotextured surfaces, such as those found on insect wings, can prevent bacterial biofilm growth (11). Nanotextured surfaces can reduce the growth of bacteria in various ways, including killing the bacteria (12), preventing initial attachment (13), or reducing cell division (14). A reduction in biofilm growth can also be found on synthetic nanostructured surfaces. Previous work from our lab has shown that a nanotextured carbon-infiltrated carbon nanotube (CICNT) surface reduces *S. aureus* biofilm growth (15). Culturing bacteria on the CICNT surface results in up to a 60%–70% reduction in viable bacteria after 36 hours compared to a bare titanium control surface.

In addition to textured surfaces, other materials can reduce the growth of bacteria. One of these materials is copper. Copper has been used as a sterilizing agent since ancient times (16). Although the full antibacterial mechanism of copper is not well understood, it is likely a multifaceted combination of bacterial membrane damage (17), reactive oxygen species production (18), and protein misfolding and aggregation (19). The antimicrobial properties of copper have recently been utilized in hospital settings on high-touch surfaces (20). Copper surfaces can greatly reduce the growth of bacteria, including *S. aureus* (21) and *P. aeruginosa* (22).

This research studies a surface that combines both a nanotextured CICNT surface and a thin copper coating. The aim of this study was to determine whether a copper-coated CICNT surface more effectively reduces bacterial biofilms than either copper or CICNT surfaces alone. It was determined that the combination of CICNT and copper resulted in a synergistic anti-biofilm effect, reducing recoverable bacteria by 99.9999% of bacteria within 12 hours. This reduction was likely due to a contact killing mechanism rather than the presence of copper ions released into the bacterial media.

## RESULTS

### Adding a small amount of copper to the CICNT surface results in a greater reduction in recoverable bacteria than copper-coated flat titanium

**Fig 1:**
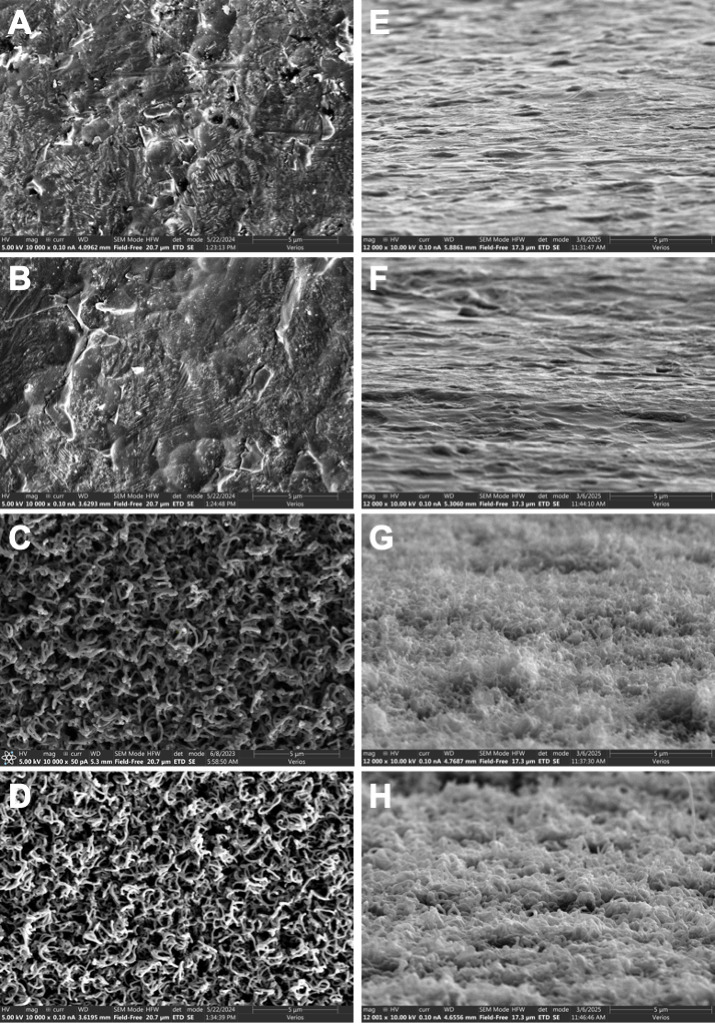
Top–down SEM images of (**A**) bare Ti, (**B**) Cu-Ti, (**C**) CICNT, and (**D**) Cu-CICNT at 10,000x before biofilm growth. Side view (80° tilt) SEM images of (**E**) bare Ti, (**F**) Cu-Ti, (**G**) CICNT, and (**H**) Cu-CICNT at 12,000x. Note that the topography remains similar after 5 nm Cu coating for both titanium and CICNT. Panel G was previously published (23), where it was also used to depict a side view of the CICNT surface.

**Fig 2:**
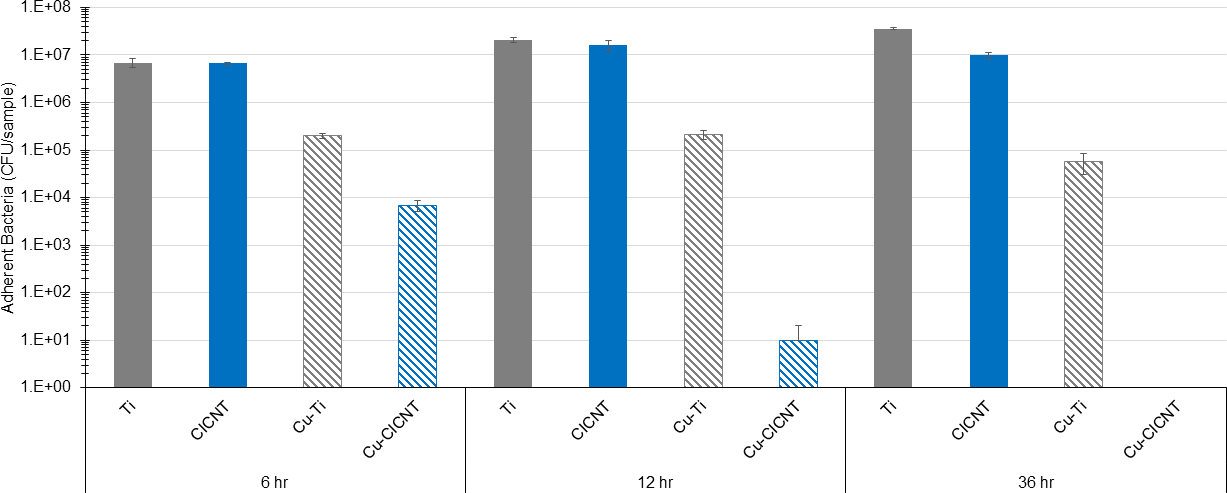
CFU/sample ± standard error for adherent *S. aureus* (JE2) grown on bare titanium, CICNT, Cu-coated Ti, or Cu-coated CICNT surfaces for the amount of time indicated. The horizontal line indicates the limit of detection. *P < 0.01, **P < 0.001, and ***P < 0.0001.

A thin film consisting of 5 nm of copper was added to the surface of both bare Ti and CICNT. The addition of the copper film did not seem to affect the surface texture (Fig. 1). To determine the effect of a copper thin film combined with the CICNT surface, RPMI inoculated with the JE2 strain of *S. aureus* (MRSA) was incubated for 6, 12, or 36 hours on bare Ti, CICNT, copper-coated Ti (Cu-Ti), and copper-coated CICNT (Cu-CICNT; Fig. 2). At 6 hours, there was no significant difference in the number of adherent bacteria retrieved from Ti or CICNT, but there was a 1.5-log (97%) reduction in the number of bacteria recovered from Cu-Ti (*P* = 0.0022) and a 3-log (99.9%) reduction in the number of bacteria recovered from the Cu-CICNT surface (*P* = 0.002, Fig. 2; Table S1 and S2). By 12 hours, there was still no significant difference in the number of adherent bacteria retrieved from Ti and CICNT, but there was a 2-log (99%) reduction in the number of bacteria recovered from Cu-Ti (*P* = 1.4×10^−6^) and a 6.3-log (99.9999%) reduction in the number of bacteria recovered from Cu-CICNT (*P* = 6.1×10^−6^, Fig. 2; Table S1 and S2). The average number of adherent bacteria recovered from the Cu-CICNT surface by 12 hours was below the limit of detection for the plate counting assay (Fig. 2). At 36 hours, there was a significant decrease in the number of bacteria on Ti and CICNT (72% reduction, *P* =< 0.0001) and a significant decrease in the number of bacteria on Cu-Ti (2.6-log (99.8%) reduction, *P* < 0.0001). At 36 hours, no bacteria were recovered from any of the Cu-CICNT samples, a 7.5-log reduction (*P* < 0.0001).

### Cell death is occurring on the Cu-CICNT surface at a greater rate than on the Ti surface

One of the limitations of a CFU-based assay is that it only quantifies living, culturable cells and cannot quantify either dead or viable but unculturable cells (24). We hypothesized that the copper surface actively kills bacteria, but to further explore this hypothesis, we used live/dead staining and flow cytometry to assess cell viability.

**Fig 3:**
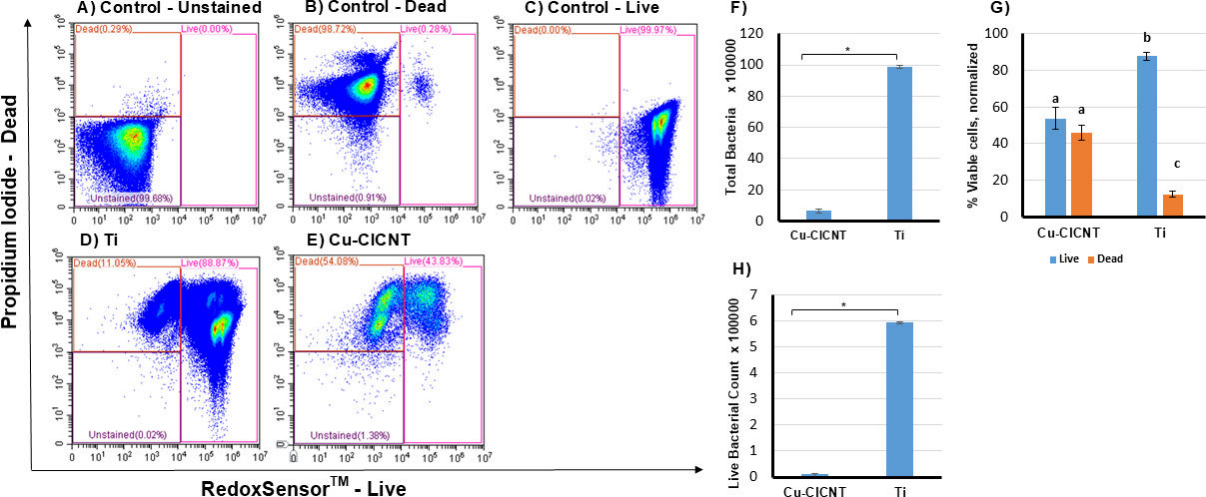
Increased bacterial cell death on Cu-CICNT compared to Ti surfaces, assessed by flow cytometry. Bacterial viability was analyzed by flow cytometry following the RedoxSensor Green and PI staining protocol, 6 hours after *S. aureus* biofilms were grown on either Ti or Cu-CICNT surfaces. (**A**) Unstained control (**B**), dead-cell positive control (CCCP-treated) (**C**), live-cell positive control (**D**), cells grown on Ti (**E**), and cells grown on Cu-CICNT. (**F**) Bar graph representing total bacteria acquired on the flow cytometer from the same volume of sample after normalization with beads. (**G**) Bar graph representing the proportion of dead and live bacterial cells, normalized with bead count, grown on either Ti or Cu-CICNT. (**H**) Live bacteria count normalized with beads. Data are presented as mean values, and error bars represent standard error from three technical replicates per condition across two independent biological experiments. Bars labeled with different letters indicate statistically significant differences (*P* < 0.05), while shared letters indicate no significant difference. The asterisk (*) indicates statistically significant differences (*P* < 0.001). The letters “a”, “b,” and “c” on panel G are used to indicate the groups that are significantly different than one another; bars labeled with different letters indicate a statistically significant difference (*P* < 0.01), while shared letters indicate no significant difference.

We grew *S. aureus* biofilms on either Ti or Cu-CICNT surfaces for 6 hours. At this time point, live bacteria were present on both surfaces (a ~ 3 log reduction on Cu-CICNT by CFU analysis), allowing us to detect both live and dead stained cells. We removed the biofilms, along with any unattached cells from each sample, and stained them with RedoxSensor Green and propidium iodide. Flow cytometry was performed using bead-based normalization to standardize event counts (Fig. 3). Significantly fewer bacteria events were detected in the same volume of samples collected from Cu-CICNT surfaces compared to Ti (14-fold difference, *P* = 0.0001, Fig. 3F). To assess the relative proportions of live and dead cells, total events were normalized such that the sum of live and dead cell percentages equaled 100% (Fig. 3G).

On the Ti surface, we found that about 12.4 ± 1.71% of recorded, bead-normalized cells stained positive for propidium iodide, indicating that the cells no longer had an intact membrane, and we categorized them as dead (Fig. 3D and G). These results are comparable to our previous findings when the cell viability was similarly assessed on CICNT without a copper coating (23). In contrast, Cu-CICNT samples exhibited a significantly higher proportion of PI+/dead cells (46.2 ± 4.10%, a fourfold increase compared to Ti, *P* = 0.0007, Fig. 3E and G). These results suggest that *bona fide* cell death occurs upon exposure to Cu-CICNT, rather than cells existing in a viable but unculturable state, which has been reported previously when bacteria are exposed to copper (25). We also found that significantly higher absolute numbers of living cells were recovered from Ti surfaces than Cu-CICNT surfaces (a ~ 50 fold difference, *P* = 0.0008, Fig. 3H).

**Fig 4:**
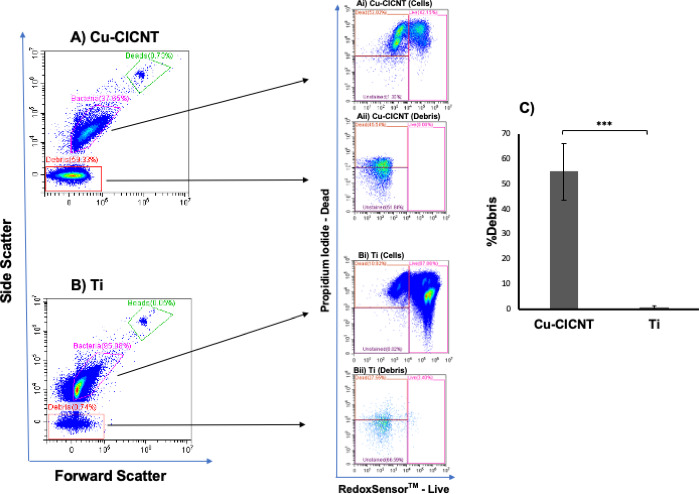
Increased bacterial debris on Cu-CICNT compared to Ti, as determined by flow cytometry. Forward scatter (FSC) and side scatter (SSC) analysis was used to identify bacterial populations and debris. (**A**) FSC and SSC of bacterial cells grown on Cu-CICNT. (Ai) Live and dead gating strategy of bacterial cells grown on Cu-CICNT. (Aii) Live and dead cell gating of debris grown on Cu-CICNT. (**B**) FSC and SSC of bacterial cells grown on Ti. (Bi) Live and dead gating of bacterial cells grown on Ti. (Bii) Live and dead cell gating of debris grown on Ti. (**C**) Bar graph representing the proportion of debris from bacterial cells grown on either Cu-CICNT or Ti. Data are presented as mean values, and error bars represent standard error from three technical replicates per condition across two independent biological experiments. The asterisk (*) indicates statistically significant differences (*P* < 0.001). All debris proportion data from each experiment are shown in Table S3.

Additionally, using an analysis of forward scatter (FSC) and side scatter (SSC) populations, we noted that significantly more debris-sized events were recorded for the Cu-CICNT surfaces than for the Ti surfaces (55.1 ± 11.2% of events vs 0.92 ± 0.62%, *P*=0.002, Fig. 4; Table S3). This could suggest that some of the bacteria on the Cu-CICNT surface have been lysed, which would result in smaller, debris-sized fragments detected by flow cytometry. These events may represent subcellular debris due to the smaller size and lower complexity. To further characterize the debris population, we gated events based on PI and RedoxSensor Green staining, as described previously. The majority of debris was either unstained or PI-positive but negative for RedoxSensor Green, suggesting that these events likely represent dead or non-viable material (Fig. 4Aii, Bii). Biofilm reduction is observed with a different isolate of *S. aureus.*

**Fig 5:**
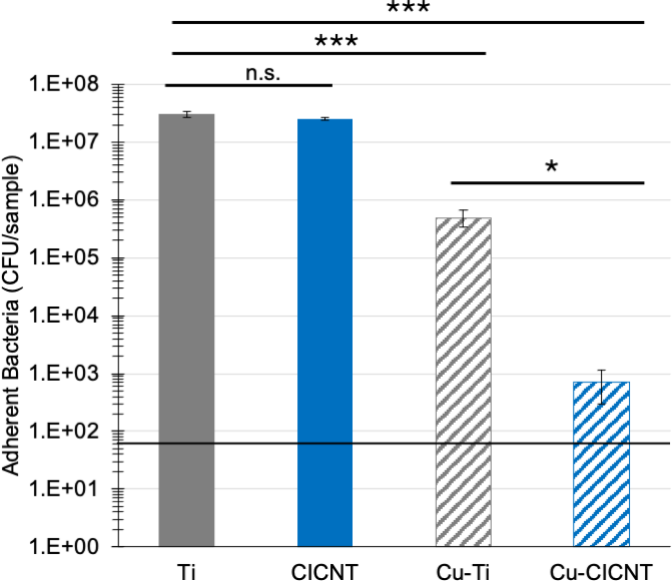
CFU/sample ± standard error for adherent *S. aureus* (SH1000) after 12 hours of growth on bare titanium, CICNT, Cu-coated Ti, or Cu-coated CICNT surfaces. Horizontal line indicates the limit of detection. *P < 0.01; ***P < 0.0001.

Different isolates of the same bacterial species may react differently to antibacterial treatments. For this reason, we tested a second isolate of *S. aureus* on the Cu-CICNT surface. RPMI inoculated with the SH1000 strain of *S. aureus* (MSSA) was incubated for 12 hours on bare Ti, CICNT, Cu-Ti, and Cu-CICNT. There was no significant difference in the number of adherent bacteria on Ti vs CICNT (*P* = 0.13), but there was a 1.7-log (98%) reduction in the number of bacteria on Cu-Ti compared to Ti (*P* = 0.00032) and a 4.6-log (99.99%) reduction on Cu-CICNT (*P* = 0.0092, Fig. 5; Table S4).

### Biofilm reduction is observed with *P. aeruginosa*

**Fig 6:**
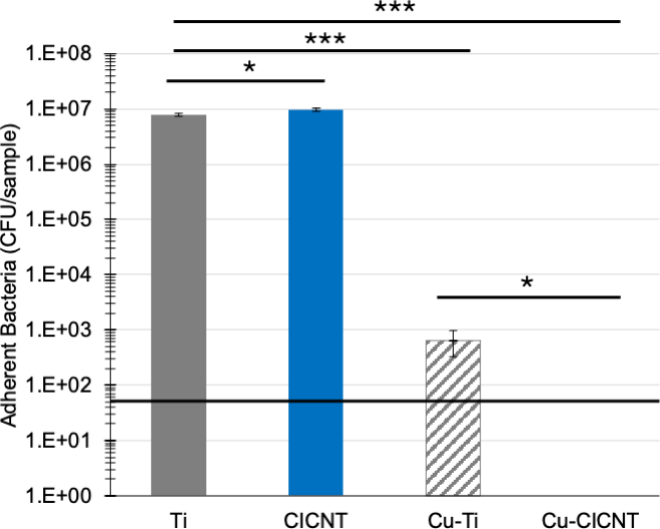
CFU/sample ± standard error for adherent *P. aeruginosa* after 12 hours of growth on bare titanium, CICNT, Cu-coated Ti, or Cu-coated CICNT surfaces. The horizontal line indicates the limit of detection.

*S. aureus* is a gram-positive bacterium. Gram-positive and gram-negative bacteria may react differently to antimicrobial surfaces due to differences in their cell walls and membrane structures (26). Gram-positive bacteria have a thick, relatively stiff layer of peptidoglycans surrounding the cells, while gram-negative bacteria have a thinner layer of peptidoglycan sandwiched between two flexible membranes. We used *P. aeruginosa* to test the response of a gram-negative bacterium to the Cu-CICNT surface since this organism is also very adept at forming biofilms on implanted devices. RPMI inoculated with strain 15442 of *P. aeruginosa* was incubated for 12 hours on bare Ti, CICNT, Cu-Ti, and Cu-CICNT. There was a small but significant difference in the number of adherent bacteria on Ti and CICNT (20% increase, *P* = 0.0087, Fig. 6). However, the differences in the number of adherent bacteria on Cu-Ti (4.2-log [99.99%] reduction, *P* = 0.037) and Cu-CICNT (6.9-log [99.9999%] reduction, *P* < 5×10^−9^) were much greater (Fig. 6; Table S5).

### The anti-biofilm properties of Cu-CICNT are in part due to an increased quantity of copper ions released into solution

Since copper is known to have antimicrobial properties, and the surface area of Cu-CICNT is expected to be much greater than for Cu-Ti, we hypothesized that growth media incubated on Cu-CICNT would have higher concentrations of released copper ions than Cu-Ti, possibly mediating the greater antimicrobial effect. The concentration of copper ions released from each surface after 12 hours of incubation was measured using inductively coupled plasma mass spectrometry (ICP-MS) and compared to a standard curve of solutions of known copper concentration. There was a 9.3% increase in the concentration of copper ions in media exposed to Cu-CICNT vs Cu-Ti. Although the increased concentration was statistically significant (*P* = 0.001), the difference in the number of ions released from the two surfaces may not be biologically relevant to the mechanism of the synergistic bacterial reduction of the Cu-CICNT surface (Table 1).

**TABLE 1:** Concentrations of copper ions released from each surface

	Copper ions (ppm) ±SE
Cu-Ti	5.65 ± 0.06
Cu-CICNT	6.20 ± 0.06

**Fig 7:**
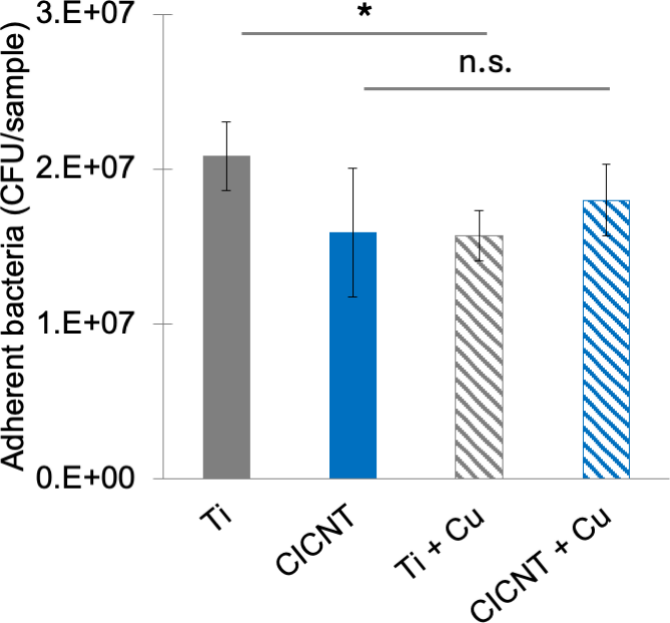
CFU/sample ± standard error for adherent *S. aureus* (JE2) grown on bare titanium, CICNT, or either surface with 6.2 ppm copper ions (from dissolved CuCl_2_) added to the culture media (Ti +Cu and CICNT +Cu). *P < 0.05, differences between all other groups are not significant.

To confirm the hypothesis that biofilm reduction was not simply due to the concentration of copper ions in the solution, 6.2 parts per million (ppm) of exogenous copper was added to the bacterial media using a CuCl_2_ solution. *S. aureus* bacteria (JE2 strain) were grown in the media containing additional copper ions on non-copper-coated Ti or CICNT for 12 hours and then enumerated. There was a small but significant decrease in adherent bacteria on Ti +copper ions vs Ti alone (25% reduction, *P* = 0.04, Fig. 7) and no significant decrease in bacteria on CICNT vs CICNT + copper ions (*P* = 0.8, Fig. 7).

**Fig 8:**
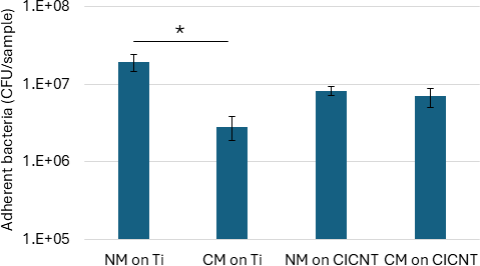
CFU/sample ± standard error for adherent *S. aureus* (JE2) grown on bare titanium. For this experiment, conditioned media (CM) was first exposed to a Cu-CICNT surface, while normal media (NM) from the same batch was used as a control. *P < 0.05.

To better understand if the anti-biofilm effect was mediated by something released into the growth media after exposure to the Cu-CICNT surface, we produced conditioned media by incubating RPMI media in the presence of Cu-CICNT for 16 hours (see Materials and Methods). This conditioned media was used to culture *S. aureus* (JE2) bacteria on a non-copper-coated Ti surface for 12 hours, alongside a Ti surface with normal media. After quantification of CFU, a significant reduction was observed in bacterial counts on Ti with conditioned media vs Ti with normal media (85.4% reduction, *P* = 0.0039, Fig. 8). This result suggests that copper ions released into solution exhibit a modest bactericidal effect, but it cannot explain the dramatic effects seen after direct exposure to a Cu-CICNT surface. Interestingly, we noted that there was no significant difference between conditioned media and normal media when cells were grown on a CICNT surface (14.9% reduction, *P* = 0.28), and this result is similar to the results in Fig. 7, where CICNT also failed to show an effect mediated by CuCl_2_ media.

## DISCUSSION

Previous work from our lab studied the effect of the CICNT surface on *S. aureus* biofilms. We found that the CICNT surface reduced the growth of *S. aureus* biofilms by 60%–80% at 36–48 hours (15, 27), but the antibiofilm effect only began after 24 hours had elapsed. This is likely why we did not see a significant difference in the number of bacteria on CICNT and Ti surfaces at the 12 hour time point in this study. The surface texture can reduce bacterial attachment and growth. Many surfaces, such as insect wings or gecko skin, have nanostructured surfaces that reduce or prevent bacterial growth (11, 28). This texturing has also been applied to synthetic materials, such as CICNT, in an attempt to replicate the antimicrobial effect.

This study aimed to understand the effect of combining the CICNT surface topography with a thin layer of copper on the biofilm formation of *S. aureus* and *P. aeruginosa,* common agents of implant-associated infection. CFU analysis demonstrated that there was a synergistic effect between the surface topography and the presence of copper, resulting in up to a 6.9-log (99.9999%) reduction in the number of bacteria present by 12 hours. This effect was seen with both an MRSA and an MSSA isolate of the gram-positive species *S. aureus* and also in the gram-negative species *P. aeruginosa*. Although the Cu-CICNT surface was highly effective against both strains of *S. aureus*, it was more effective against the JE2 strain than against the SH1000 strain. JE2 is an MRSA strain, and SH1000 is an MSSA strain, but studies show that copper surfaces are highly effective against both MRSA and MSSA isolates (29). The SH1000 strain has also been shown to form stronger biofilms than the JE2 strain (30). A stronger biofilm could potentially slow the copper anti-biofilm effect (31), which may explain why SH1000 biofilms were not reduced to the same extent as JE2 biofilms.

Copper has been studied as an antimicrobial surface for many years. Copper coatings can add an antimicrobial effect to materials with other desirable material properties (32). For example, one study found that coating nanotextured stainless steel with 6 µm of copper resulted in a 2-log reduction in the number of bacteria on the surface after 24 hours of incubation (33). Another group found that steel surfaces coated in a 20–40-μm-thick layer of copper resulted in a 4-log reduction in the number of *S. aureus* and *E. coli* on the surface after 24 hours of incubation (34). For comparison, our results showed a range of a 4.6-log reduction (*S. aureus* strain SH1000) to a 6.9-log reduction for *P. aeruginosa* strain 15442. Most of the surfaces in these other reports used much thicker layers of copper than our Cu-CICNT surface, which was coated with only 5 nm of copper, representing a copper surface over 1,000-fold thinner than these other reports. Copper is a costly material that can cause complications to eukaryotic tissues at high concentrations (35), so a thinner layer is preferable. A thicker layer of copper would also obscure the nanotextured surface of the CICNT, potentially eliminating a synergistic effect between the surface texture and copper.

The combination of nanotextured surfaces and copper could result in improved bacterial killing. For instance, different methods of copper processing result in different surface textures, and one study found that electroporated copper surfaces killed bacteria more effectively than either polished or native rolled copper, indicating that both surface texture and the presence of copper affect bacterial killing (36). However, not all studies show a synergistic effect between surface texture and the presence of copper (37, 38). By contrast, our Cu-CICNT surface, with only a 5 nm layer of copper-coated nanotubes, reduced recoverable bacteria by up to 6.9 logs in only 12 hours, significantly more than a flat 5 nm copper-coated titanium surface.

We made a number of observations that indicate that exposure to the Cu-CICNT surface results in cell killing. First, we observed a 14-fold reduction in the total number of cell events recovered from the Cu-CICNT vs Ti surface via flow cytometry. Analysis of bacterial viability using RedoxSensor Green and propidium iodide (PI) staining revealed a fourfold increase in the proportion of dead (PI^+^) cells associated with Cu-CICNT surfaces relative to Ti. Strikingly, we observed a 60-fold increase in subcellular debris from Cu-CICNT samples, which remained largely unstained by RedoxSensor Green stain, suggesting that this material represented nonviable, fragmented bacterial remnants. This population exhibited a distinct FSC/SSC signature profile consistent with bacterial debris, as previously characterized (39), and is distinguishable from the bacterial population based on size and cellular complexity.

In addition, the number of viable cells recovered from Cu-CICNT surfaces was markedly reduced compared to Ti surfaces (50-fold reduction). These findings suggest that exposure to the Cu-CICNT surface results in bacterial cell death and potentially results in cell fragmentation. Importantly, this observed reduction in viable bacteria on the Cu-CICNT surface is not merely due to detachment from the surface, as both attached and unattached bacteria were analyzed. While these results do not entirely rule out the presence of viable but nonculturable bacteria, they do suggest that cell death is taking place. Future studies will further investigate the potential existence of viable but non-culturable cells on Cu-CICNT surfaces.

To better understand the mechanism of bacterial reduction by the Cu-CICNT surface, we quantified the copper ions released from the surface in 12 hours using ICP-MS. ICP-MS uses a high-temperature plasma atomizer to ionize the sample, followed by analysis of the ion beam by the mass-charge ratio. Consequently, a measurement of copper ions by ICP-MS is a measure of total copper ions and does not differentiate between free and complexed copper. Although a significant difference in the number of copper ions released was noted between the two surfaces, the difference in the number of ions released was likely too small to account for the large differences in the number of bacteria recovered from the two surfaces. The maximum value of copper ions released after 12 hours was 6.197 ppm or 0.098 mM.

The minimum inhibitory concentration (MIC) for copper ions in solution depends on several factors, including the exact bacterial isolate and the culture media composition. For planktonic cells (SH1000 strain of *S. aureus*) in a rich medium (TSB or LB), growth was uninhibited by up to 1 mM copper and was completely inhibited by 10 mM (40). In rich media, bacteria can tolerate greater concentrations of copper ions because they are chelated by media components. In RPMI, some examples of these components are L-cysteine and glutathione, which both complex with copper (41, 42). One study found that in RPMI (without FBS supplementation), planktonic *S. aureus* strain JE2 had an MIC of 0.5 mM copper (43). In our study, we measured 6.197 ppm copper ions released from the Cu-CICNT surface (0.098 mM). This is well below the MIC found in the studies referenced above. These results suggest that the amount of copper ions in solution is likely not sufficient to explain the rate of bacterial reduction on either Cu-Ti or Cu-CICNT.

We sought to further develop this hypothesis by adding an equivalent number of exogenous copper ions to the bacterial culture media and growing on non-copper-coated surfaces for 12 hours. There was a significant decrease in bacteria on Ti vs Ti + copper ions (1.33-fold, 25%); it was not a large decrease and likely cannot account for the multi-log reduction in bacteria on copper-coated Ti or CICNT. There was no significant decrease in recoverable bacteria on CICNT vs CICNT + copper ions. The oxidation state of copper ions released from Cu-CICNT may differ from that of CuCl_2_, which could change observed antimicrobial effects, so we conducted an additional experiment where we grew biofilms using conditioned media that had been exposed to Cu-CICNT. This experiment similarly showed a significant reduction of attached cells in the presence of copper-containing conditioned media, and interestingly, the percent reduction was greater than when testing CuCl_2_ media (85.4% vs 25%, respectively). These results suggest that differences exist between CuCl_2_-prepared media and conditioned media. We did not explore the oxidation state of either media, which could explain the difference. Interestingly, both CuCl_2_-generated media and conditioned media failed to show a difference on CICNT surfaces; it was only seen on Ti surfaces. We surmised that CICNT may chelate copper ions, thus reducing the effectiveness of copper in the presence of carbon, and several studies indicate that carbon is able to remove copper from solution (44, 45), and this knowledge has intentionally been used to remove copper from wastewater (46). Overall, our results suggest that bacterial contact with the textured Cu-CICNT surface itself is responsible for the massive reduction in recoverable bacteria on that surface. We propose that Cu-CICNT has a combined mechanism of action, where both soluble copper ions and direct contact-dependent killing—due to surface topography—contribute to the enhanced bactericidal activity of Cu-CICNT surfaces.

One limitation of this study is that it did not distinguish between different oxidation states of free copper or copper-ligand complexes. Instead, total copper ions were quantified by ICP-MS, and then an equivalent number was added to the culture media, some of which then could interact with media components. Future work, including the use of x-ray spectroscopic analysis of the surfaces and chelation of specific copper species, will be done to better understand the exact mechanisms at play and whether particular copper species are largely responsible for the synergistic antibacterial effect.

Other studies have also shown that contact killing, rather than copper ions in solution, is largely responsible for bacterial cell death on copper surfaces (47, 48). One study developed a copper surface that could be altered by the addition of a fine, mesh-like polymer grid that prevented direct bacterial contact with the copper but allowed copper ions to freely escape into the solution. When bacteria were grown on the copper surface without the barrier, they were killed. When they could grow in the presence of the copper ions but not in direct contact with the surface, bacterial killing did not occur (49).

One current hypothesis for the general mechanism of copper contact killing is through a combination of membrane damage and intracellular damage (29, 50). Copper hydroxides derived from the copper surface may be able to react with the peptides that crosslink peptidoglycans (51). Once the peptidoglycan lattice is sufficiently degraded, copper hydroxides may be able to contact and react with the lipid membrane, resulting in cell lysis (51). Other intracellular mechanisms may also be involved (50). The Cu-CICNT surface likely kills bacteria with a contact-killing mechanism similar to the one described above, with the addition of minor but significant stresses exerted by the topography on adhered cells, making the bacteria more susceptible to copper contact killing. This combination resulted in a synergistic effect—up to a 6.9-log reduction in viable bacteria compared to uncoated CICNT and up to a 4.3-log reduction in viable bacteria compared to flat copper. Understanding these combined effects could provide valuable insights into the development of antimicrobial surfaces and guide future research on optimizing copper-based bactericidal materials.

## MATERIALS AND METHODS

### CICNT and control sample preparation

Surfaces coated in carbon-infiltrated carbon nanotubes were prepared as described previously (15). Briefly, medical-grade Ti6Al4V alloy (Ti) was cut from sheet stock into 9 mm squares (herein referred to as samples). Samples were sonicated in isopropyl alcohol for 15 minutes, rinsed in deionized water, and dried. For the creation of CICNT samples, 200 nm of Al_2_O_3_ was deposited on the surface of each sample as a barrier layer using electron-beam deposition. This was followed by the deposition of a 6 nm thin film of iron as a catalyst layer using a thermal evaporator.

The prepared samples were then placed into a tube furnace for CICNT growth, which was performed as described previously (15), with the infiltration step resulting in nanotubes with a final diameter of approximately 150 nm, which was confirmed using scanning electron microscopy (SEM).

### Copper-coated sample preparation

Five nanometers of copper was deposited on both bare Ti and CICNT samples using a thermal evaporator. Filament-based thermal evaporation is a form of physical vapor deposition in which an electric current runs through the crucible containing the evaporation source in a vacuum chamber. The current heats the evaporation source until it vaporizes. These vaporized atoms can then condense onto a material overhead, forming a thin film coating. To prepare copper-coated samples, a voltage of 120 V was used to generate a deposition rate of about 5 Å per second until 5 nm of copper had been deposited on the surface of each sample. This addition of copper did not affect the surface topography of either type of sample (see Fig. 1 for SEM images).

### Bacterial strains

JE2 (BEI Resources NR-46543) is derived from USA300 LAC, a well-characterized methicillin-resistant *S. aureus* (MRSA) strain isolated from a patient from the Los Angeles County jail in 2002 (52). JE2 differs from the parent LAC strain by the removal of two cryptic plasmids (53). SH1000 (BEI Resources NR-55396) is a methicillin-sensitive human isolate of *S. aureus* (MSSA) with the *rbs*U gene restored. 15442 is a *Pseudomonas aeruginosa* strain commonly used in pharmacological testing that was originally isolated from a water bottle in a laboratory animal room.

### Bacterial culture

Cultures of *S. aureus* were grown in shaking culture at 200 rpm for 16–18 hours in TSB. Cultures of *P. aeruginosa* were grown in shaking culture at 200 rpm for 16–18 hours in Luria-Bertani broth (LB). All cultures were then diluted to an optical density at 600 nm of 0.02 in RPMI 1640 media (10^6^ CFU), supplemented with sodium bicarbonate and HEPES (4-(2-hydroxyethyl)−1-piperazineethanesulfonic acid), as well as 2% heat-inactivated fetal bovine serum (FBS). Since pH plays a role in the solubility of copper ions, the pH was adjusted to 7.3 before sterile filtering the media. A CuCl_2_ solution was used to add the appropriate amount of copper to the prepared RPMI media for experiments with exogenous copper ions. Twenty-five microliters of inoculated RPMI was pipetted as a droplet onto the top of each sample, as previously reported (15). The droplets of bacterial culture were then incubated on the surface of each sample at 37°C for the given amount of time. Each experiment was repeated at least three times, with each independent experiment being performed on at least two separate samples of each type.

### Quantification of bacteria in biofilms

Colony-forming unit (CFU) analysis was performed as reported previously (15). Briefly, after incubation, the samples were washed once in sterile 1 x phosphate-buffered saline (PBS) to remove non-adherent cells. Samples were then removed to a clean well. Five hundred microliters of PBS was added to the sample surface and pipetted vigorously to dislodge the adherent cells in the biofilm (we have previously shown by scanning electron microscopy that this method is highly effective at removing biofilm cells (15)). Once the cells from the biofilm were suspended in the PBS, 10 µL from each well was then removed and serially diluted in PBS before being spread on LB agar plates. The plates were then incubated at 37 C for 24 hours, colonies were counted, and CFU counts were calculated. Because of the use of serial dilutions to get countable plates, the limit of detection of the assay was determined to be 50 CFU since 10 µL was removed from 500 µL for the first dilution.

To prepare the conditioned media, 500 µL of RPMI media rested on top of a copper-coated CICNT sample for 16 hours at 37 C in a CO_2_ incubator. Normal media from the same batch was used for comparison purposes but without incubation on copper-coated CICNT. When testing the conditioned media, biofilms were grown under the same conditions as above. CFU analyses were also conducted and quantified the same as above.

### Copper ion quantification

The concentration of copper ions released from both copper-coated titanium and copper-coated CICNT was quantified using inductively coupled plasma mass spectrometry (ICP-MS, Agilent ICP-MS 7800). Five hundred microliters of sterile RPMI 1640, supplemented as described above, was incubated on the surface of each sample for 12 hours. After 12 hours, the medium was removed and diluted 10-fold for ICP-MS analysis. The copper content of each sample was determined by comparing it to a standard curve based on dilutions of a copper standard.

### Bacterial viability assessment using the BacLight RedoxSensor Green Vitality Kit and flow cytometry

Bacterial viability was assessed using the BacLight RedoxSensor Green Vitality Kit (Thermo Fisher Scientific, USA), which consists of two key reagents: propidium iodide (PI) and RedoxSensor Green (RSG). PI (20 mM in DMSO) is excluded by bacterial cells with intact membranes but penetrates bacteria with compromised membranes, binding to DNA and thus serving as a marker of non-viable or dead bacterial cells. In contrast, RedoxSensor Green reagent stain (1 mM in DMSO, and diluted 1:10 in PBS prior to use) penetrates live bacterial cells and fluoresces green if the bacteria are alive.

Using this method, biofilms were grown on samples for 6 hours and then disrupted by vigorous pipetting with 500 µL PBS, as previously described. As a positive control for membrane-compromised cells, 2 µL of the electron transport chain uncoupler carbon cyanide m-chlorophenyl hydrazone (CCCP, 10 µM) was added to approximately 10^6^ healthy bacteria in 500 µL of PBS, followed by a 5 minute incubation at room temperature. Healthy untreated bacteria served as live, membrane-intact controls. Unstained controls were also used to facilitate compensation and gating.

For staining, 1 µL RedoxSensor Green reagent and 1 µL of PI were added to each 500 µL bacterial suspension, gently vortexed, and incubated at room temperature in the dark for 5 minutes. Immediately before analysis, counting beads (Thermo Fisher Scientific, USA) were added to each sample to normalize volume acquisition. Samples were analyzed using a CytoFLEX flow cytometer (Beckman Coulter, Brea, CA, USA).

### Flow cytometry gating strategy and identification of debris

Gating was performed using the positive, negative, and unstained controls according to the manufacturer’s guidelines. Bacterial populations were initially identified using forward scatter (FSC) and side scatter (SSC) on logarithmic scales. FSC was used to assess cell size, while SSC indicated internal complexity. Debris was identified as events with markedly lower FSC and SSC values, falling outside the main bacterial population gate. Data were processed and analyzed using FlowJo software (version 10.6.2).

### Statistical analysis

Data were analyzed using Student’s *t*-test. Statistically significant differences were attributed to variables with *P* ≤ 0.05.

## SUPPLEMENTAL MATERIAL

Supplemental tables aem.01053-25-s0001.docx Tables S1 to S5.

ASM does not own the copyrights to Supplemental Material that may be linked to, or accessed through, an article. The authors have granted ASM a non-exclusive, world-wide license to publish the Supplemental Material files. Please contact the corresponding author directly for reuse.
