# Supplementary material for: Copper-coated carbon-infiltrated carbon nanotube surfaces effectively inhibit Staphylococcus aureus and Pseudomonas aeruginosa biofilm formation
Source: Appl Environ Microbiol. 2025 Jul 8;91(8):e01053-25. doi: 10.1128/aem.01053-25 (PMC12366356; doi:10.1128/aem.01053-25)
Supplement: Supplemental tables — Tables S1 to S5. [file aem.01053-25-s0001.docx]

Supplementary Table 1: *P*-values from a statistical comparison of the number of adherent bacteria (*S. aureus* isolate JE2) found on the following surfaces: Ti, CICNT, Cu-Ti, and Cu-CICNT.

| **6 hours** | | | |
| --- | --- | --- | --- |
|  | **CICNT** | **Cu-Ti** | **Cu-CICNT** |
| **Ti** | 0.44 | 0.0022 | 0.0020 |
| **CICNT** | - | 5.8 x 10^-6^ | 5.2 x 10^-6^ |
| **Cu-Ti** |  | - | 1.1 x 10^-7^ |
|  |  |  |  |
| **12 hours** | | | |
|  | **CICNT** | **Cu-Ti** | **Cu-CICNT** |
| **Ti** | 0.16 | 1.4 x 10^-6^ | 6.1 x 10^-6^ |
| **CICNT** | - | 0.0018 | 0.0035 |
| **Cu-Ti** |  | - | 0.0013 |
| **36 hours** | | | |
|  | **CICNT** | **Cu-Ti** | **Cu-CICNT** |
| **Ti** | 8.9 x 10^-11^ | 5.6 x 10^-12^ | 5.4 x 10^-12^ |
| **CICNT** | - | 8.0 x 10^-5^ | 7.3 x 10^-5^ |
| **Cu-Ti** |  | - | 0.020 |

Supplementary Table 2: Log difference in the number of adherent bacteria on various surfaces (*S. aureus* isolate JE2)

| **6 hours** | | | |
| --- | --- | --- | --- |
|  | **CICNT** | **Cu-Ti** | **Cu-CICNT** |
| **Ti** | 0.44 | 0.0022 | 0.0020 |
| **CICNT** | - | 5.8 x 10^-6^ | 5.2 x 10^-6^ |
| **Cu-Ti** |  | - | 1.1 x 10^-7^ |
|  |  |  |  |
| **12 hours** | | | |
|  | **CICNT** | **Cu-Ti** | **Cu-CICNT** |
| **Ti** | 0.16 | 1.4 x 10^-6^ | 6.1 x 10^-6^ |
| **CICNT** | - | 0.0018 | 0.0035 |
| **Cu-Ti** |  | - | 0.0013 |
| **36 hours** | | | |
|  | **CICNT** | **Cu-Ti** | **Cu-CICNT** |
| **Ti** | 8.9 x 10^-11^ | 5.6 x 10^-12^ | 5.4 x 10^-12^ |
| **CICNT** | - | 8.0 x 10^-5^ | 7.3 x 10^-5^ |
| **Cu-Ti** |  | - | 0.020 |

Supplementary Table 3: Proportion of debris from bacterial cells grown on either Cu-CICNT or Ti samples (each entry is an individual sample).

| **Surface** | **% Debris** |
| --- | --- |
| Cu-CICNT 1 | 0.02% |
| Cu-CICNT 2 | 81.26% |
| Cu-CICNT 3 | 56.46% |
| Cu-CICNT 4 | 44.02% |
| Cu-CICNT 5 | 34.59% |
| Cu-CICNT 6 | 59.33% |
| Ti 1 | 0.00% |
| Ti 2 | 0.00% |
| Ti 3 | 0.00% |
| Ti 4 | 3.82% |
| Ti 5 | 0.74% |
| Ti 6 | 0.03% |

Supplementary Table 4: (Above) *P*-values from a statistical comparison of the number of adherent bacteria found on various surfaces from SH1000 after 12 hours of incubation. (Below) Log difference in the number of adherent bacteria (SH1000) on various surfaces after 12 hours of incubation.

| ***S. aureus* (SH1000) *P*-values** | | | | |
| --- | --- | --- | --- | --- |
|  | **Ti** | **CICNT** | **Cu-Ti** | **Cu-CICNT** |
| **Ti** | - | 0.13 | 0.00035 | 0.00032 |
| **CICNT** |  | - | 7.0 x 10^-6^ | 7.3 x 10^-6^ |
| **Cu-Ti** |  |  | - | 0.0092 |
| **Cu-CICNT** |  |  |  | - |
|  |  |  |  |  |
| **Log Difference** | | | | |
|  | **Ti** | **CICNT** | **Cu-Ti** | **Cu-CICNT** |
| **Ti** | - | 0.082 | 1.79 | 4.63 |
| **CICNT** |  | - | 1.71 | 4.55 |
| **Cu-Ti** |  |  | - | 2.84 |
| **Cu-CICNT** |  |  |  | - |

Supplementary Table 5: (Above) *P*-values from a statistical comparison of the number of adherent bacteria found on various surfaces from *P. aeruginosa* after 12 hours of incubation. (Below) Log difference in the number of adherent bacteria *(P. aeruginosa)* on various surfaces after 12 hours of incubation.

| ***P. aeruginosa* (15442) *P-*values** | | | | |
| --- | --- | --- | --- | --- |
|  | **Ti** | **CICNT** | **Cu-Ti** | **Cu-CICNT** |
| **Ti** | - | 0.0087 | 5.3 x 10^-9^ | 5.3 x 10^-9^ |
| **CICNT** |  | - | 2.9 x 10^-9^ | 2.9 x 10^-9^ |
| **Cu-Ti** |  |  | - | 0.037 |
| **Cu-CICNT** |  |  |  | - |
|  |  |  |  |  |
| **Log Difference** | | | | |
|  | **Ti** | **CICNT** | **Cu-Ti** | **Cu-CICNT** |
| **Ti** | - | 0.10 | 4.08 | 6.89 |
| **CICNT** |  | - | 4.18 | 6.99 |
| **Cu-Ti** |  |  | - | 2.81 |
| **Cu-CICNT** |  |  |  | - |
